# Transcriptomics and metabolomics provide insight into the anti-browning mechanism of selenium in freshly cut apples

**DOI:** 10.3389/fpls.2023.1176936

**Published:** 2023-05-08

**Authors:** Xinyue Wang, Xuemei Zhang, Peng Jia, Haoan Luan, Guohui Qi, Han Li, Suping Guo

**Affiliations:** College of Forestry, Hebei Agricultural University, Baoding, China

**Keywords:** anti-browning, fresh-cut, apple, transcriptome, metabolome

## Abstract

Enzymatic browning has a considerable negative impact on the acceptability and marketability of freshly cut apples. However, the molecular mechanism by which selenium (Se) positively affects freshly cut apples in this regard is not yet clear. In this study, 0.75 kg/plant of Se-enriched organic fertilizer was applied to “Fuji” apple trees during the young fruit stage (M_5_, May 25), the early fruit enlargement stage (M_6_, June 25), and the fruit enlargement stage (M_7_, July 25), respectively. The same amount of Se-free organic fertilizer was applied as a control. Herein, the regulatory mechanism by which exogenous Se exerts its anti-browning effect in freshly cut apples was investigated. The results showed that the M_7_ treatment applied in Se-reinforced apples could remarkably inhibit their browning at 1 h after being freshly cut. Additionally, the expression of polyphenol oxidase (*PPO*) and peroxidase (*POD*) genes treated with exogenous Se was significantly reduced compared to controls. Moreover, the lipoxygenase (*LOX*) and phospholipase D (*PLD*) genes, which are involved in membrane lipid oxidation, were expressed at higher levels in the control. The gene expression levels of the antioxidant enzymes catalase (*CAT*), superoxide dismutase (*SOD*), glutathione S-transferase (*GST*), and ascorbate peroxidase (*APX*) were upregulated in the different exogenous Se treatment groups. Similarly, the main metabolites measured during the browning process were phenols and lipids; thus, it could be speculated that the mechanism by which exogenous Se produces its anti-browning effect may be by reducing phenolase activity, improving the antioxidant capacity of the fruits, and alleviating membrane lipid peroxidation. In summary, this study provides evidence regarding and insight into the response mechanism employed by exogenous Se to inhibit browning in freshly cut apples.

## 1 Introduction

Apple (*Malus domestica* Borkh.) is considered one of the four major fruits in the world and features a rich nutritional profile, including high contents of vitamins, minerals, polyphenols, and flavonoids (Bondonno et al., 2017). With the continued improvement and ongoing changes occurring in modern living standards, lifestyles, and consumption habits, consumer demand for clean, fresh, convenience foods that feature high nutritional value and are free of additives has fleetly increased in tandem (Yousuf et al., 2020; Hu et al., 2021). Owing to the above characteristics of freshly cut apples, the future market potential for freshly cut apple products is predicted to increase significantly. Freshly cut apples have become a popular snack food in fast food restaurants, school lunch programs, and fruit stores in recent years (Sumonsiri, 2017; Fan, 2022). However, the processing steps used on apples, such as peeling and cutting, cause damage to the fruit tissue. In turn, this renders them prone to browning, thereby seriously affecting the quality and nutritional value of the final product (Mishra et al., 2012). Because of this, produce browning represents a major global challenge to address in order to improve the fresh-cutting and product processing of various fruits and vegetables, as their browning represents a major cause of reduced appearance, nutritional value, and shortened shelf life (Hamdan et al., 2022; Zha et al., 2022). Therefore, exploring a labor-saving, efficient, and safe anti-browning method for fruits and vegetables, as well as clarifying the mechanism underlying its occurrence, has emerged as an economically important scientific problem to be solved.

Browning reactions can be divided into two categories: enzymatic browning and non-enzymatic browning (Moon et al., 2020; Zuo et al., 2021). Enzymatic browning is considered the main reaction affecting freshly cut product browning, and refers to the phenomenon by which polyphenol oxidase (PPO) and peroxidase (POD) catalyze the oxidation of phenolic compounds to colorless quinones. Subsequently, these quinones polymerize to form melanin. These substances make the product appear brown, red, or black (Yousuf et al., 2020). Fruit or vegetable lesions lead to membrane lipid peroxidation and the disruption of membrane structure, thereby causing an imbalance in the production and scavenging of reactive oxygen species (ROS) (Imahori, 2014; Kong et al., 2020). Previous studies have found that the occurrence of browning in “Nanguo pear” is associated with damage to the cell membrane system (Wang et al., 2018). Phospholipase D (PLD) and lipoxygenase (LOX), which are involved in membrane lipid oxidation reactions, are key enzymes associated with enzymatic browning (Singh et al., 2018; Xiao et al., 2021). Phenolic substances are important substrates for the enzymatic browning of fruits and vegetables. Polyphenols that are prevalent in apples include phenolic acids, such as chlorogenic acid and coumaroylquinic acid, and flavonoids, such as epicatechin, phlorizin, and quercetin (Putnik et al., 2017). Studies have also shown that apple polyphenols confer apples with strong antioxidant activity (Kähkönen et al., 1999; Boyer and Liu, 2004). Mitigating browning by changing the substrate concentration is difficult. At present, existing research on strategies to alleviate the browning of fruits and vegetables focuses on post-harvest treatments that inhibit the activity of the PPO enzyme, where mainly physical and chemical measures have been adopted (i.e., the use of packaging technologies, high and low temperatures, high-pressure conditions, ultraviolet irradiation, and the addition of related enzyme inhibitors, among others) (Qi, 2017; Moon et al., 2020). However, these feature several drawbacks, including requiring complex procedures, cost increases, and potential security risks. Until now, there has been a lack of systematic research conducted on the anti-browning of freshly cut apples after absorbing selenium (Se) during the cultivation process. Therefore, such a strategy involving processing by Se enrichment may be of high utility in solving the existing issue of freshly cut apple browning.

Se is an essential trace element required for normal human physiological metabolism. Its various physiological functions include the scavenging of ROS, maintaining the structural integrity of cell membranes, contributing to antioxidant capacity, and delaying cell senescence (Nielsen, 1990; Zhang, 2006; Kamran et al., 2019). Currently, Se deficiency is a problem affecting 1 billion people across roughly 40 countries worldwide (Ullah et al., 2019; Wang et al., 2020). China is one such country affected by serious Se deficiency, in which Se-deficient provinces account for more than 70% of the total area of the country, while the per-capita intake of Se is only 44.4 μg/day, which is lower than the recommended level (55–400 μg/day) set by the World Health Organization (WHO) (Liao et al., 2021). Se can be better absorbed by the human body after being converted into highly bioavailable Se by plants. Developing diversified Se-rich agricultural products therefore represents a viable means to assist Chinese residents in meeting their nutritional and health needs (Tang et al., 2019). It has been reported that Se application can increase the Se content of barley by three to five times (Korkman, 1980) and significantly improve the fruit quality of apples (Liu et al., 2020), cherries (Qi et al., 2019), grapes (Zhu et al., 2007), and persimmons (Yang et al., 2013), among other fruits.

With the development of high-throughput sequencing, transcriptomic and metabolomic analysis methods have been widely used to study gene and metabolite expression profiles and identify key functional pathways in plants, thus revealing the molecular mechanisms involved in specific biological processes (Zhou et al., 2016; Hao et al., 2018). For example, the molecular mechanism involved in the post-cutting browning of potato varieties with different browning sensitivities has previously been elucidated by transcriptomics and non-targeted metabolomics (Qiao et al., 2022). Using RNA sequencing technology, Chen C. et al. (2021) found that the expression of most differentially expressed genes (DEGs) associated with the antioxidant system was upregulated after H_2_S treatment in freshly cut apples, while the expression levels of genes encoding PPO, POD, and LOX were suppressed, thus successfully elucidating the mechanism of browning inhibition in freshly cut apples treated with H_2_S. Tang et al. (2020) employed metabolomics to find that the levels of hyperoside, POD, superoxide dismutase (SOD), and ascorbic acid, as well as the gene expression levels of related genes (*MdPAL*, *MdCHS*, *MdCHI*, *MdANS*, and *MdFLS*) were lower in browning-sensitive apples.

In this study, a combined transcriptomic and metabolomic approach was used to investigate the response mechanism to exogenous Se anti-browning in freshly cut apples. Differentially accumulated metabolites, DEGs, and differences in signaling pathways between exogenously Se-treated and control apples after cutting (0 h and 1 h) were thus derived. The findings contribute new insight into the mechanism by which exogenous Se treatment inhibits browning in freshly cut apples.

## 2 Materials and methods

### 2.1 Experimental design and sample collection

The experiment was conducted in Gangdi Village, Neiqiu County, Xingtai City, Hebei Province (37°28′N, 114°52′E) from March to November 2021, which is located in the eastern foothills of the southern Taihang Mountain gneiss mountainous area and resides at an altitude of 562–1,200 m; the average annual temperature is 11.6°C, the average annual precipitation is 684.8 mm, and the frost-free period is 180 days. The row spacing of the apple trees used for collection was 3 m × 1.5 m. The main cultivar was “2001Fuji”, and the pollination cultivar was Honglu. Healthy 8-year-old apple trees at full fruit stages were selected. A 0.75-kg weight of Se-enriched organic fertilizer was applied per plant at the young fruit stage (M_5_, May 25), early fruit expansion stage (M_6_, June 25), and fruit expansion stage (M_7_, July 25), respectively. Note: Se in this study refers to amino acid-chelated Se; its content was ≥1,000 mg kg^−1^, the mass fraction of organic matter was ≥45%, and the mass fraction of the total nutrients (nitrogen + phosphorus pentoxide + potassium oxide) was ≥5.0%. The ditch fertilization method (three trees per repeat, three repeats, and 36 trees in total) was used. The test site conditions were the same with a consistent management level. The control trees were treated with organic fertilizer that was identical to the experimental fertilizer except for being free of Se.

Samples of “Fuji” apples were collected at commercial maturity on 23 October 2021. Apples were selected in different directions around the canopy periphery based on being well-grown, evenly shaped, and without mechanical damage. After harvest, the fruits were immediately transported to the laboratory and stored at 4°C before the experiments were conducted. The apples were washed gently under running water to remove any surface dirt and were dried naturally. A portion of fresh fruit samples was used for the determination of the surface color of freshly cut apples. Some fruits were sliced into 0.3- to 0.5-cm-thick slices with a sterile knife, using nine slices per treatment, each from a different apple. We collected samples of “Fuji” apple fruit flesh at two time points after cutting (0 h and 1 h) for transcriptomic and metabolite analyses; three biological replicates were collected for the transcriptomic analysis, while six biological replicates were collected for metabolome analysis. All samples were immediately frozen in liquid nitrogen and stored at −80°C.

### 2.2 Measurement of browning index and color difference

Fruit color was analyzed as previously described (Xu et al., 2022). Each selected fruit was cut lengthwise into two pieces with a sterile knife to measure the surface color of the freshly cut fruit. The L*, a*, and b* values were measured and recorded by a colorimeter (CR-400, Konica Minolta Inc., Japan) at a random position selected on the freshly cut surface of the fruit along a time gradient (0 h, 0.5 h, 1 h, 2 h, 4 h, and 24 h). Each treatment group was measured five times. The browning index (BI) was calculated using Eq. (1), while the color difference (Δ*E*) was calculated using Eq. (3).


(1) BI=[100(X−0.31)]/0.172

where *X* was calculated using Eq. (2).


(2) $$X=\frac{\left(a^{*}+1.75L^{*}\right)}{\left(5.645L^{*}+a^{*}-3.012b^{*}\right)}$$

(3) $$\mathrm{\Delta E}={\left[{\left({\mathrm{\Delta L}}^{*}\right)}^{2}+{\left({\mathrm{\Delta a}}^{*}\right)}^{2}+{\left({\mathrm{\Delta b}}^{*}\right)}^{2}\right]}^{1/2}$$

where *L* ^*^ is the brightness value, *a* ^*^ is the red–green degree value, and *b* ^*^ is the yellow–blue degree value.

### 2.3 Transcriptomic analysis of post-cut “Fuji” apples

#### 2.3.1 RNA extraction and RNA quantification

Total RNA was extracted from the frozen pulp tissue of apples (control and M_7_) using TRIzol® reagent according to the manufacturer’s instructions (Invitrogen) and genomic DNA was removed using DNase I (Takara). Then, the quality of the obtained RNA was determined by a 2100 Bioanalyzer (Agilent Technologies Inc., USA) and quantified using the ND-2000 (NanoDrop Technologies). A high-quality RNA sample (OD260/280 = 1.8–2.2, OD260/230 ≥ 2.0, RIN ≥ 8.0, 28S:18S ≥ 1.0 > 1 μg) was used to construct the sequencing library.

#### 2.3.2 RNA-seq transcriptomic analysis and differentially expressed gene identification

RNA purification, reverse transcription, library construction, and sequencing were performed at Shanghai Majorbio Bio-pharm Biotechnology Co., Ltd. (Shanghai, China). The transcriptome library was prepared following the TruSeq™ RNA Sample Preparation Kit from Illumina (San Diego, CA) using 1 μg of total RNA. Messenger RNA was isolated according to the polyA selection method by oligo (dT) beads and then fragmented by fragmentation buffer. Subsequently, double-stranded cDNA was synthesized using a Superscript Double-Stranded cDNA Synthesis Kit (Invitrogen, CA) with random hexamer primers (Illumina). Then, the synthesized cDNA was subjected to end-repair, phosphorylation, and “A” base addition. Libraries were size-selected for cDNA target fragments of 300 bp on 2% Low Range Ultra Agarose followed by PCR amplification using Phusion DNA polymerase (NEB) for 15 PCR cycles. After being quantified by TBS380, the paired-end RNA-seq sequencing library was sequenced with the Illumina NovaSeq 6000 sequencer (2 × 150 bp read length). After Illumina sequencing, the clean data were compared with the reference genome (https://iris.angers.inra.fr/gddh13/the-apple-genome-Downloads.html). To identify DEGs between two different samples, the expression level of each gene was calculated according to the transcripts per million reads (TPM) method. Finally, GO (http://www.geneontology.org) and KEGG (http://www.genome.jp/kegg/pathway.html) were used to conduct a biological functional classification analysis of the DEGs. Essentially, differential expression analysis was performed using DESeq2, where DEGs with |log_2_ (foldchange)| ≥ 1 and *p*-adjust< 0.05 were considered to denote significantly different expression levels (Min et al., 2019; Zuo et al., 2021).

### 2.4 Enzyme activity measurements

Apple fruit samples were ground in liquid nitrogen before being measured for their activities of the contained enzymes PPO (BC0190), POD (BC0090), LOX (BC0320), PLD (BC2415), SOD (BC0170), catalase (CAT) (BC0205), ascorbate peroxidase (APX) (BC0220), and glutathione S-transferase (GST) (BC0350) using the corresponding assay kits (Solarbio Inc. Beijing, China) in accordance with the manufacturer’s instructions. The activities of PPO, POD, LOX, PLD, SOD, CAT, APX, and GST were determined by a spectrophotometer (UV-Vis 1700, Shimadzu, Columbia, MD, USA) at 410 nm, 470 nm, 280 nm, 290 nm, 560 nm, 240 nm, 510 nm, and 340 nm, respectively. Enzyme activity units (U) were expressed as U g^−1^ per fresh weight (FW). All experiments were performed with three independent biological replicates.

### 2.5 Quantitative real−time PCR

To validate the RNA-seq data, the expression levels of 16 selected DEGs were quantified using quantitative real−time PCR (qRT-PCR) following the manufacturer’s protocols (PrimeScript™ 1st strand cDNA Synthesis Kit, Takara, Japan). Total RNA was extracted from three independent biological replicates of both the control and the treated M_7_ apple material before being purified by the TaKaRa MiniBEST Plant RNA Extraction Kit (TaKaRa, Japan). The primers used for qRT-PCR are listed in **Table 1**. Each qRT-PCR reaction contained 20 µl of RNase-free dH_2_O, 0.4 µl of 10 μM of each of the forward and reverse primers, 10 µl of 2 × SYBR real-time PCR premixture, and 1 µl of diluted cDNA. The amplification program was initiated by one cycle of 95°C for 5 min, then followed by 40 cycles composed of 95°C for 15 s and 60°C for 30 s, and was finally completed with a melting curve analysis procedure. The relative expression level was calculated using the comparative 2^−ΔΔCT^ method (Livak and Schmittgen, 2001). Three biological replicates were used for the qRT-PCR analysis.

**Table 1:** Primers used in qRT-PCR for the validation of RNA-Seq data.

Gene name	Forward primer sequence	Reverse primer sequence
**Actin**	TATGTTGCTATCCAGGCCGT	GTAAGATCACGCCCAGCAAG
**MD15G1106600**	TTCTCACCCCTCGTCTTCAC	GCTGGGATTGGACGAAACTC
**MD03G1289900**	CGTGAACCGGAAGCGATTAG	GTAGCACCTCCTCCTCTCAC
**MD03G1296100**	GTTACAGAGACAGCGTGCAG	CACTTCTGTCAGCCGCATAC
**MD13G1092700**	CCGCCATTGATGAAGCTCTT	TGACTCTCCACAGCTTCGAG
**MD15G1071400**	ATTTGAGAACGAGGCCTCCA	GCAAGCATGATGAGGCAGAT
**MD12G1181000**	CATGGAGAACAGCGGGATTG	GAAGGAGGCTGGAGTGACTT
**MD02G1148800**	GGACGAGTGGATGGATAGCA	AACAGGGATTGCTGAGTCGA
**MD08G1057400**	TGTTTGGGCAAGAAGTGAGC	TAGTCAAGTCGGCGAAGGAG
**MD07G1022600**	GCTTTGGAGGTTATGCAGGG	CCCCAAAAGCAACGAGTCAA
**MD08G1106900**	ACCAACAGCAGCAAAGGAAG	AGGCGCTTCGAAACAACAAA
**MD12G1040800**	GTTACAAGAACGGCCAGGTG	CCGCGATTTGATTCTCCGTT
**MD07G1222900**	TCCACTGCTTCACTTGAGGT	ATGGCCTTCCTTCTTGAGCT
**MD00G1144300**	CAAGGGCAAGTTGGTTCGAA	ATTCCCCAGTGACCCTTCTG
**MD16G1093500**	AATTCCACGGGATCCTCGAA	TACCTTCTCCGCCGTCTTTT
**MD10G1143400**	TTCCTCCAGAACGACTTCCC	CACATCGCCCTTGATCACAG
**MD13G1090900**	TCTTGTTTGGTTGAAGCCGG	ACAATCACGGCCTTGCATTT

### 2.6 Metabolomic analysis of post-cutting “Fuji” apples

#### 2.6.1 Metabolite extraction

Metabolomic analysis was performed according to previously published methods with slight modifications (Qiao et al., 2022). Frozen pulp tissue (50 mg) was accurately weighed before its metabolites were extracted using a 400-µl methanol:water (4:1, v/v) solution, with 0.02 mg/ml L-2-chlorophenylalanin as the internal standard. The mixture was allowed to settle at −10°C before being treated by the Wonbio-96c high-throughput tissue crusher (Shanghai Wanbo Biotechnology Co., Ltd.) at 50 Hz for 6 min, which was followed by ultrasound at 40 kHz for 30 min at 5°C. The samples were subsequently placed at −20°C for 30 min to precipitate the contained proteins. After centrifugation at 13,000 *g* and 4°C for 15 min, the supernatant was carefully transferred to sample vials for LC-MS analysis. As a part of the system conditioning and quality control process, a pooled quality control sample (QC) was prepared by mixing equal volumes of all samples. The QC samples were disposed of and tested in the same manner as the analytic samples.

#### 2.6.2 LC-MS/MS analysis

The instrument platform used for LC-MS analysis was the UHPLC-Q Exactive HF-X system (Thermo Fisher Scientific). The chromatographic conditions were as follows: 2 μl of sample was separated by an HSS T3 column (100 mm × 2.1 mm i.d., 1.8 μm; Waters, Milford, USA) and then underwent mass spectrometry detection. The mobile phases consisted of 0.1% formic acid in water:acetonitrile (95:5, v/v) (solvent A) and 0.1% formic acid in acetonitrile:isopropanol:water (47.5:47.5:5, v/v) (solvent B). To equilibrate the systems, the solvent gradient was altered according to the following timescale: 0% B–24.5% B (0.4 ml/min) over 0–3.5 min; 24.5% B–65% B (0.4 ml/min) over 3.5–5 min; 65% B–100% B (0.4 ml/min) over 5–5.5 min; 100% B–100% B (0.4 ml/min to 0.6 ml/min) over 5.5–7.4 min; 100% B–51.5% B (0.6 ml/min) over 7.4–7.6 min; 51.5% B–0% B (0.6 ml/min to 0.5 ml/min) over 7.4–7.6 min; 0% B–0% B (0.5 ml/min to 0.4 ml/min) over 7.8–9 min; and 0% B–0% B (0.4 ml/min) over 9–10 min. The sample injection volume was 2 µl and the flow rate was set to 0.4 ml/min. The column temperature was maintained at 40°C. During the analysis period, all samples were stored at 4°C.

The mass spectrometric data were collected using a Thermo UHPLC-Q Exactive HF-X Mass Spectrometer equipped with an electrospray ionization (ESI) source. The optimal conditions were set as follows: heater temperature, 425°C; capillary temperature, 325°C; sheath gas flow rate, 50 Arb; aux gas flow rate, 13 Arb; ion-spray voltage floating (ISVF), −3,500 V in negative mode and 3,500 V in positive mode; normalized collision energy, 20–40–60 V rolling for MS/MS. The full MS resolution was 60,000 and the MS/MS resolution was 7,500. Data acquisition was performed using the data-dependent acquisition (DDA) mode. The detection was carried out over a mass range of 70–1,050 m/z.

#### 2.6.3 LC/MS data preprocessing

The data were analyzed through the free online platform of Majorbio Cloud Platform (cloud.majorbio.com). To reduce the incidence of errors caused by sample preparation and instrument instability, the response intensity of the sample mass spectrum peaks was normalized by the sum normalization method, and the normalized data matrix was obtained. Furthermore, variables with a relative standard deviation (RSD) > 30% of that of the QC samples were removed, and log_10_ logarithmization was performed to obtain the final data matrix for subsequent analysis.

Variance analysis was performed on the matrix file after data preprocessing. The R package “ropls” (Version 1.6.2) was used to perform principal component analysis (PCA) and orthogonal least partial squares discriminant analysis (OPLS-DA). In addition, the Student’s *t*-test and fold difference analysis were performed. The selection of significantly differential metabolites was determined based on the variable importance in the projection (VIP) obtained by the OPLS-DA model and the *p*-value of the Student’s *t*-test. Metabolites with VIP > 1 and *p<* 0.05 were considered significantly differential metabolites.

### 2.7 Statistical analysis

Each sample had three to six replicates. The data were analyzed by SPSS 24 software (Chicago, USA). Pairwise comparisons were performed using the *t*-test, while multiple comparisons were performed using analysis of variance (ANOVA) tests. *p<* 0.05 was adopted to indicate significant differences. The differences between multiple groups were represented by a, b, c, and d. The same letter indicated no difference, while different letters indicated significant differences (*p<* 0.05). All the data were expressed as the means ± standard deviations (SDs).

## 3 Results

### 3.1 Changes in the browning degree of freshly cut “Fuji” apples

**Figure 1:**
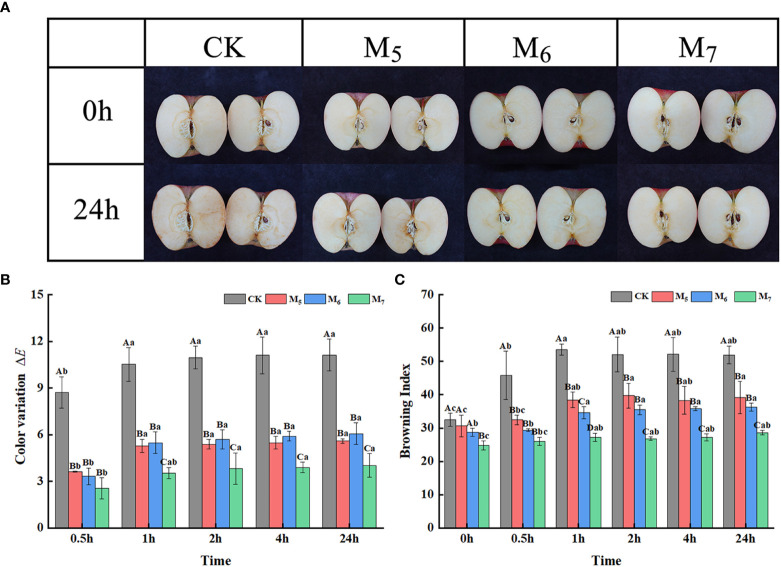
Exogenous selenium (Se) inhibited the browning in fresh-cut apples. **(A)** Browning phenotype of fresh-cut apple at 0 h and 1 h. **(B)** Comparison of the BI of fresh-cut apple fruit at different time points. **(C)** Comparison of the Δ*E* of fresh-cut apple fruit at different time points. CK: application of non-Se-enriched organic fertilizer; M_5_: application of Se fertilizer at the young fruit stage (May 25); M_6_: application of Se fertilizer at the early fruit expansion stage (June 25); M_7_: application of Se fertilizer at the fruit expansion stage (July 25). Uppercase letters indicate significant differences between CK, M_5_, M_6_, and M_7_ at the same time point, and lowercase letters indicate significant differences between different time points for the same treatment (*p<* 0.05). Vertical bars indicate the standard error of the means and values are the mean of three replicates.

Among the samples, the fruit treated with exogenous Se in M_7_ exhibited an outstanding anti-browning effect when compared to the control 1 h after being freshly cut (**Figure 1A**). The Δ*E* value represents the degree of change in the surface color of the freshly cut apples at different time intervals when compared with that of 0 h; a higher Δ*E* value indicates a more substantial degree of browning (Wen et al., 2020). With the extension of time after being freshly cut, the Δ*E* values of the Se treatment group apples at different stages were significantly lower than those of the control (**Figure 1B**). Freshly cut apples were left to brown for 24 h, after which, the Δ*E* values of fruit in the control and the ones treated with exogenous Se in M_7_ were 11.1 and 4.0, respectively, rendering the control group value 2.8 times that of the Se M_7_ group.

The BI values were found to be consistent with the Δ*E* values. The BI values of each treatment group were significantly lower than that of the control (**Figure 1C**). The BI values of the control group and the group treated with exogenous Se in M_7_ apples were 51.9 (maximum) and 28.6 (minimum), respectively. The maximum change in the browning degree across different treatment groups occurred within 1 h after being freshly cut, while then there was no significant change observed in the degree of browning between the treatments. Therefore, apples at 0 h and 1 h after being freshly cut in the control and M_7_ groups were used as further research materials in this experiment.

### 3.2 Transcriptome gene expression analysis

**Figure 2:**
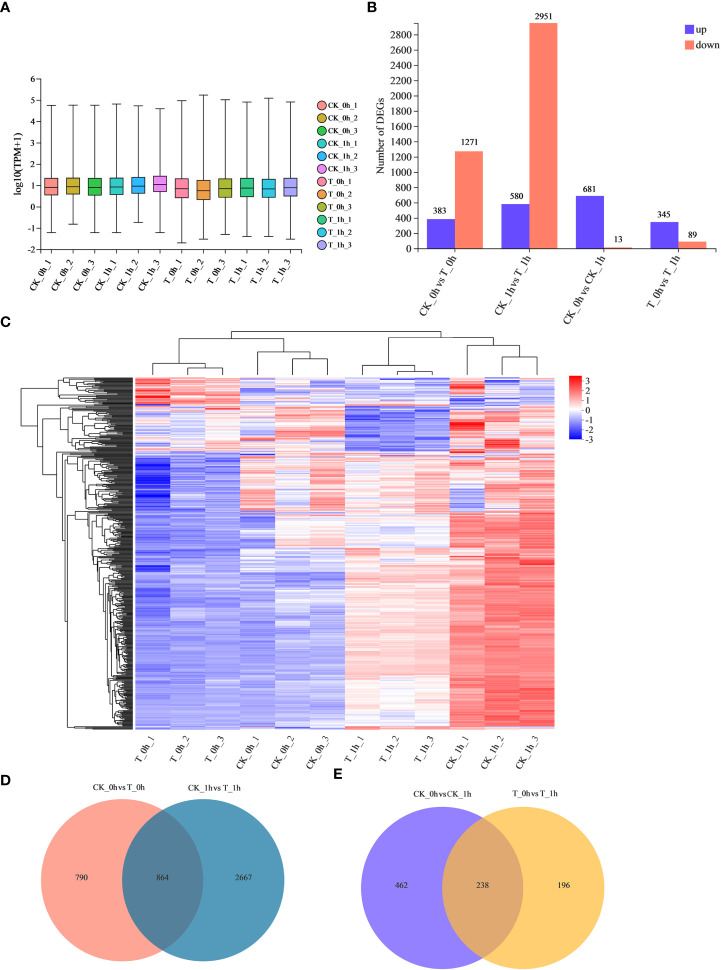
Analysis of identified DEGs during browning of fresh-cut apple fruits. **(A)** Total gene expression in each sample based on log_10_ TPM values. **(B)** Numbers of up- and downregulated DEGs in CK_0h vs. T_0h, CK_1h vs. T_1h, CK_0h vs. CK_1h, and T_0h vs. T_1h. **(C)** Fresh-cut apple fruit samples clustering heatmap. Red represents upregulated genes; blue represents downregulated genes. **(D)** Venn diagrams show unique and common DEGs in CK_0h vs. T_0h and CK_1h vs. T_1h. **(E)** Venn diagrams show the unique and common DEGs in CK_0h vs. CK_1h and T_0h vs. T_1h.

To investigate the molecular mechanism underpinning the effectiveness of exogenous Se treatment in inhibiting browning in freshly cut apples, RNA-seq libraries were designed, including control (CK) samples and M_7_ exogenously Se-treated (T) samples at 0 h and 1 h. An average of 44.9 million clean reads were obtained per sample (**Table 2**), with alignment rates ranging from 88.8% to 94.0%, which were uniquely mapped to the apple genome (**Supplementary Table 1**). Hierarchical clustering of the samples based on the number of transcripts per kilobase of exon model per million mapped reads (TPM) showed that all the biological replicates clustered together, suggesting a high reliability of our sequencing data (**Figure 2C**). We also analyzed the distribution of the gene expression levels in each sample. Symmetry and dispersion of the data distribution were observed with very high levels of gene expression (**Figure 2A**).

**Table 2:** Quality summary of sequencing data.

Sample	Raw reads	Clean reads	Q20 (%)	Q30 (%)	GC content (%)
**CK_0h_1**	44,919,722	44,219,480	98.12	94.65	47.96
**CK_0h_2**	42,747,048	42,123,178	98.18	94.8	47.9
**CK_0h_3**	45,782,610	44,982,890	97.89	94.07	47.89
**CK_1h_1**	43,192,562	42,384,512	98.02	94.39	48.07
**CK_1h_2**	45,627,600	45,057,108	98.16	94.75	47.75
**CK_1h_3**	45,634,172	45,027,390	98.17	94.79	47.68
**T_0h_1**	45,114,754	44,475,026	98.08	94.56	48.29
**T_0h_2**	44,149,462	43,500,522	98.17	94.8	47.85
**T_0h_3**	46,059,084	45,412,730	98.1	94.59	48.17
**T_1h_1**	48,748,498	48,179,570	98.1	94.59	48.31
**T_1h_2**	48,708,482	48,140,042	98.09	94.57	48.02
**T_1h_3**	46,222,498	45,710,538	98.11	94.6	48.20

We identified 1,654 (383 upregulated and 1,271 downregulated) and 3,531 (580 upregulated and 2,951 downregulated) DEGs in the CK_0h vs. T_0h and CK_1h vs. T_1h groups, respectively, with a total of 864 genes in common (**Figures 2B, D**; **Supplementary Table 2**). There were more DEGs in CK_1h vs. T_1h than those counted in CK_0h vs. T_0h, indicating that a majority of the transcriptional responses manifested within the post-cutting period. In the control, there were 681 and 13 DEGs found to be up- and downregulated at 1 h compared to 0 h, respectively (**Figure 2B**). In the T group, 345 and 89 DEGs were up- and downregulated, respectively, after 1 h of storage, with only 238 genes in common (**Figures 2B, E**). Thus, CK vs. T had a greater number of DEGs. Exogenous Se treatment altered the gene expression patterns of the freshly cut apples during storage, which may be related to its browning inhibition effect.

#### 3.2.1 Functional annotation and classification of DEGs

**Figure 3:**
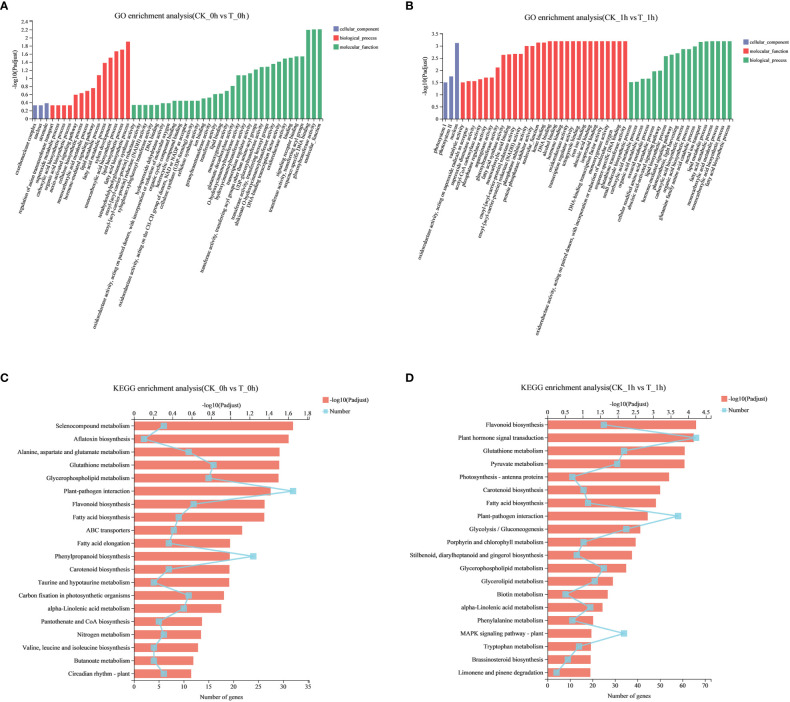
Functional enrichment analysis of DEGs during browning of fresh-cut apple fruits. **(A)** GO functional enrichment of the CK_0h vs. T_0h. **(B)** GO functional enrichment of the CK_1h vs. T_1h. **(C)** KEGG enrichment of the CK_0h vs. T_0h. **(D)** KEGG enrichment of the CK_1h vs. T_1h.

Functional enrichment analysis is an effective method to explore the biological functions of DEGs. According to GO analysis, 1,154 and 3,958 DEGs in the apples at 0 h and 1 h after being freshly cut, respectively, were assigned to three main GO functional categories. In CK_0h vs. T_0h, the DEGs were divided into 206 functional groups (**Supplementary Table 3**); cellular components, biological processes, and molecular functions accounted for 16, 93, and 97 terms, respectively. In CK_1h vs. T_1h, DEGs were divided into 276 functional groups (**Supplementary Table 4**); cellular components, biological processes, and molecular functions accounted for 10, 147, and 119 terms, respectively. GO analysis showed that the DEGs at 0 h and 1 h were mainly enriched in the two categories of biological processes and molecular functions. The top 50 enriched functional groups at 0 h and 1 h were selected (**Figures 3A, B**), and the dominant groups in the main categories of biological processes were fatty acid biosynthesis processes, lipid biosynthesis processes, and monocarboxylic acid biosynthesis processes. Oxidoreductase activity, glutathione transferase activity, and DNA-binding transcription factor activity were clustered in molecular functions.

To further identify the important metabolic pathways involved in browning, we used KEGG pathway analysis to determine the biological functions of the DEGs. Among the KEGG enrichment pathways at 0 h and 1 h, there were 107 and 122 significant pathways identified (**Supplementary Tables 5, 6**), respectively (*p<* 0.05). Enrichment analysis of the top 20 KEGG pathways indicated that most of the identified DEGs were involved in fatty acid biosynthesis (Ko00061); alanine, aspartate, and glutamate metabolism (Ko00250); glutathione metabolism (Ko00480); plant–pathogen interactions (Ko04626); phenylpropanoid biosynthesis (Ko00940); and flavonoid biosynthesis (Ko00941) (**Figures 3C, D**).

#### 3.2.2 Differential expression of browning-related genes in freshly cut apples

**Figure 4:**
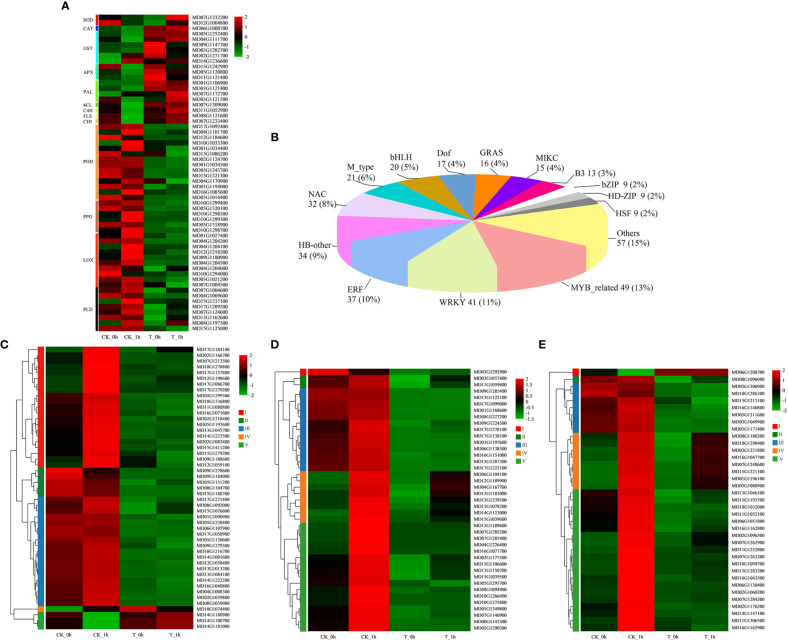
Differential expression of genes and transcription factors associated with browning in fresh-cut apples. **(A)** Differential expression of 59 browning-related genes under control and exogenous Se treatment. **(B)** Number of differentially expressed transcription factors. **(C)** Heatmap of DEGs encoding transcription factors (MYB). **(D)** Heatmap of DEGs encoding transcription factors (WRKY). **(E)** Heatmap of DEGs encoding transcription factors (ERF). Red represents upregulated genes; green represents downregulated genes.

We detected transcriptomic changes that occurred in important enrichment pathways associated with apple browning after apple cutting by conducting a clustering analysis of gene expression patterns. These changes were then illustrated in a heatmap (**Figure 4A**; **Supplementary Table 7**). We analyzed the DEGs related to oxidoreductase activities, such as SOD, CAT, POD, and PPO, as well as the activities of GST and APX in the glutathione metabolic pathway, containing 2, 1, 14, 6, 6, and 3 coding genes, respectively.

Compared to 0 h after being freshly cut, three PPO-coding genes were upregulated in the control at 1 h after being freshly cut (MD10G1298500, with a 1.8 log_2_FC significant increase; MD10G1299300, with a 2.6 log_2_FC significant increase; MD10G1298700, with a 1.3log_2_FC significant increase), but no significant changes in PPO expression were observed in the exogenous Se treatment apples at 0 h and 1 h after being freshly cut. *PPO* gene expression was significantly reduced at T_0h (MD05G1318900; a 4.2 log_2_FC significant reduction) and T_1h (MD10G1299400; a 6.9 log_2_FC significant reduction) compared to the control. The PPO activity of the control samples at 1 h after being freshly cut was higher than that at 0 h, while the PPO activity of the exogenous Se-treated samples was consistently lower than that of the control. In CK_0h vs. CK_1h, three POD-coding genes (MD04G1101700, with a 2.6 log_2_FC significant increase; MD10G1033300, with a 3.6 log_2_FC significant increase; MD16G1085600, with a 2.6 log_2_FC significant increase) were found to have upregulated expression levels in CK_1h, while one POD-coding gene (MD12G1184600; a 2.2 log_2_FC significant increase) was upregulated in CK_0h. Compared with CK_0h, one POD-encoding gene (MD17G1092400; a 6.3 log_2_FC significant reduction) was significantly downregulated in T_0h. Compared with CK_1h, one POD-coding gene (MD04G1101700; a 2.5 log_2_FC significant reduction) was significantly downregulated in T_1h.

Exogenous Se treatment induced the increased expression of genes encoding GST, and compared with CK_0h, five *GST* genes were upregulated in T_0h (such as MD09G1147300, with a 1.3 log_2_FC significant increase, and MD03G1282700, with a 1.3 log_2_FC significant increase). Compared with CK_1h, two *GST* genes were upregulated in T_1h (MD05G1252400, with a 1.3 log_2_FC significant increase, and MD04G1111700, with a 3.4 log_2_FC significant increase). Compared to 0 h after being freshly cut, four and two *GSTs* were downregulated and upregulated at 1 h, respectively. In addition, one CAT-coding gene showed a higher expression level in the exogenous Se treatment group compared to the control. Furthermore, the two *APX* genes showed significantly higher expression levels in T_0h compared to CK_0h, while one SOD-coding gene was significantly upregulated in CK_0h and T_1h compared to T_0h and CK_1h.

In addition, we observed significant differences in the expression levels of genes encoding LOX and PLD in the DEGs associated with fatty acid and lipid biosynthesis pathways for the control and exogenous Se-treated samples, containing 10 and 8 coding genes, respectively. In the control, the following was observed: seven *LOX* genes (such as MD09G1180900; a 2.5 log_2_FC significant increase) and four *PLD* genes (such as MD15G1237100; a 5.8 log_2_FC significant increase) were upregulated at 0 h to 1 h after being freshly cut; two *LOX* and two *PLD* genes were upregulated 0 h after being freshly cut; one *LOX* gene and one *PLD* gene were upregulated at 0 h and 1 h after being freshly cut. These *LOX* and *PLD* genes were almost downregulated at 0 h and 1 h after being freshly cut in the exogenous Se treatment.

Four genes encoding phenylalanine ammonia-lyase (PAL) and one gene encoding 4-coumarate-CoA ligase (4CL) were identified in the phenylpropanoid biosynthetic pathway, all of which were found to have higher expression levels in the T group compared to the control group, while two genes encoding PAL had higher expression levels at T_0h and T_1h, respectively. Similarly, in the flavonoid biosynthetic pathway, a cinnamate-4-hydroxylase gene (*C4H*), a flavonol synthase (*FLS*) gene, and a chalcone-flavanone isomerase gene (*CHI*) were upregulated in the exogenous Se treatment at 1 h after being freshly cut relative to 0 h, but in the control group, these genes showed the reverse trend.

#### 3.2.3 Differential expression of transcription factor genes in apples after being freshly cut

To investigate the effect of exogenous Se treatment on the browning of freshly cut apples, we identified a family of transcription factor DEGs in apples from two treatment groups (0 h and 1 h after cutting). A total of 379 transcription factor genes from different families (i.e., MYB, WRKY, ERF, HN-other, and NAC families) were differentially expressed during the browning process (**Figure 4B**; **Supplementary Table 8**). The two transcription factor families with the most DEGs were found to be MYB (49; 13%), WRKY (41; 11%), and ERF (37; 10%) (**Figure 4B**). These differentially expressed transcription factor genes were classified into five categories according to their expression levels in the groups CK_0h, CK_1h, T_0h, and T_1h. Among the 49 MYB genes, the expression levels of type I, type II, and type III genes were found to be significantly higher in the CK group than in the T group, with 1 h showing significantly higher upregulation than 0 h for the type I genes in the CK group (**Figure 4C**). The remaining MYB genes (type IV and type V) exhibited the opposite expression pattern. The first three types of the WRKY family genes exhibited higher levels of gene expression in CK compared to T, while the type IV and V genes were downregulated in the other three groups, except for CK_1h, which was upregulated (**Figure 4D**). Similarly, the five types of genes in the ERF family had essentially the same expression pattern as the WRKY family genes (**Figure 4E**).

### 3.3 Analysis of enzyme activity related to browning of “Fuji” apples

**Figure 5:**
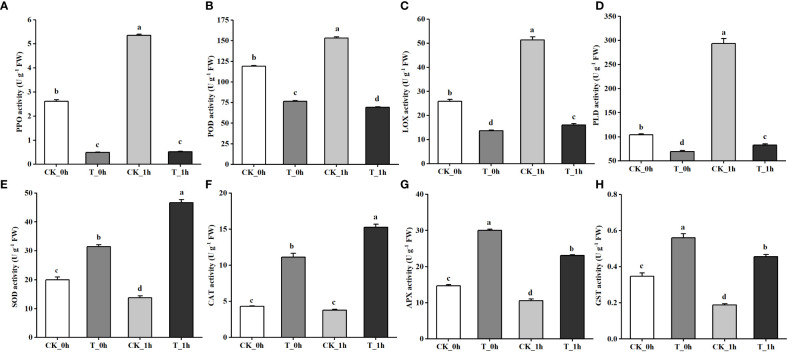
Physiological indexes of control and Se-treated apples at 0 and 1 h after cutting. **(A)** PPO activity. **(B)** POD activity. **(C)** LOX activity. **(D)** PLD activity. **(E)** SOD activity. **(F)** PPO activity. **(G)** APX activity. **(H)** GST activity. The different letters for each same sampling point indicate significantly different at *p<* 0.05 according to Duncan’s multiple range test. Vertical bars indicate the standard error of the means and values are the mean of three replicates.

From the transcriptomic results, it was found that exogenous Se treatment inhibited browning in the freshly cut apples due to changes in several key genes. Therefore, we measured the activities of key enzymes involved in browning, including PPO, POD, LOX, and PLD, as well as the activities of several key enzymes involved in anti-browning, including SOD, CAT, APX, and GST, as shown in **Figures 5A–H**, respectively. The activities of PPO, POD, LOX, and PLD in the control group were significantly higher than those in the Se treatment group, and the enzyme activities were higher at CK_1h than at CK_0h. The activities of the four antioxidant enzymes showed that their expression levels in the Se treatment group were significantly higher than in the control group. SOD and CAT activities were higher at T_1h compared to T_0h, while the activities of APX and GST were higher at T_0h. The changing trends of these physiological indexes were consistent with the transcriptomic results.

### 3.4 Validation of DEGs by qRT-PCR

**Figure 6:**
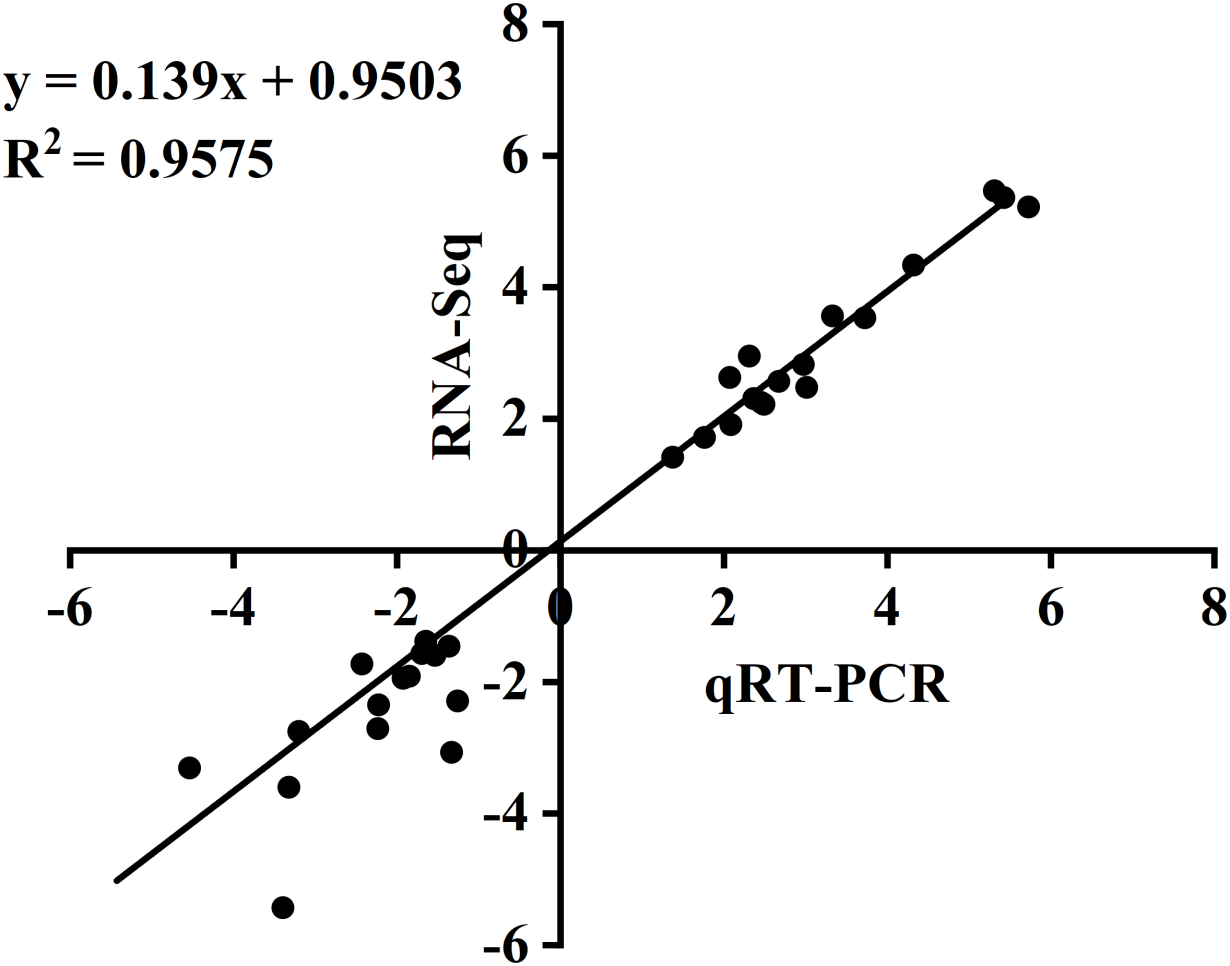
Correlation analysis between RNA-seq and qRT-PCR data.

To validate the RNA-seq results, we performed qRT-PCR expression analysis on 16 apple genes. There was no significant difference between qRT-PCR analysis results and RNA-seq data and similar trends were observed in up- and downregulated genes (**Figure 6**). These results confirm the reliability of RNA-seq results and reflect the actual transcriptome changes in this study.

### 3.5 Differentially abundant metabolites during the browning of “Fuji” apples

**Figure 7:**
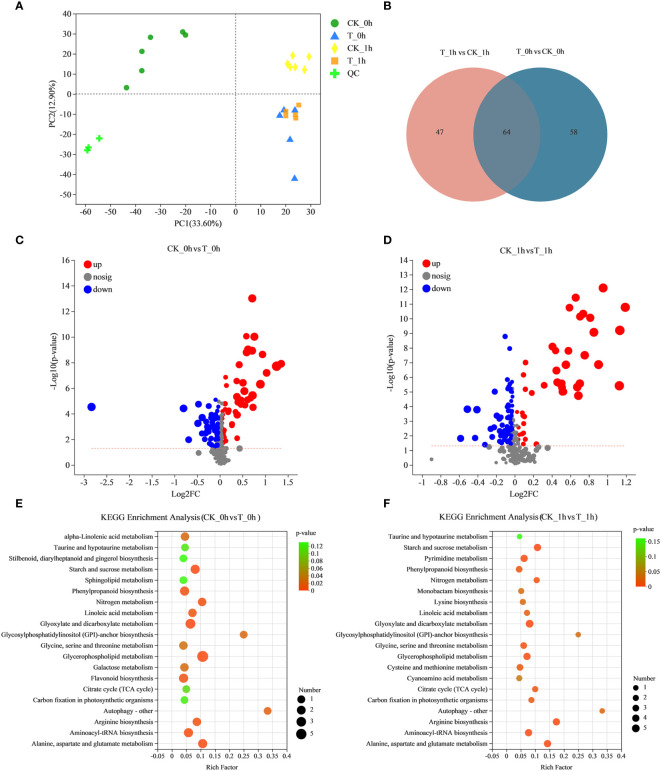
Analysis of metabolite accumulation during the browning of fresh-cut apple fruit. **(A)** PCA analysis of metabolite content in all samples. **(B)** Venn diagrams showing common DAMs in CK_0h vs. T_0h and CK_1h vs. T_1h. **(C)** Volcano map of fresh-cut apple fruit browning (CK_0h vs. T_0h). The abscissa presents the log_2_ (fold-change), whereas the ordinate presents the −log_10_ (*p*-value). **(D)** Volcano map of fresh-cut apple fruit browning (CK_1h vs. T_1h). The abscissa presents the log_2_ (fold-change), whereas the ordinate presents the −log_10_ (*p*-value). **(E)** Statistics of KEGG enrichment in fresh-cut apple fruits under control and exogenous Se treatment (CK_0h vs. T_0h). **(F)** Statistics of KEGG enrichment in fresh-cut apple fruits under control and exogenous Se treatment (CK_0h vs. T_0h).

To investigate the significant changes that occurred in the metabolites of freshly cut apples with exogenous Se treatment corresponding to the anti-browning effect, differential accumulation metabolites (DAMs) were analyzed for CK_0h vs. T_0h and CK_1h vs. T_1h. The PCA of the metabolic quantification of 24 fruit samples showed that all the biological replicates clustered together, suggesting a good correlation between replicates and the high reliability of our data (**Figure 7A**). Interestingly, we observed a clear separation between CK_0h and CK_1h, suggesting that the metabolite profiles of these two samples were distinctly different. However, there was no clear separation between T_0h and T_1h, suggesting that the metabolite profiles of these two samples were similar. Se treatment may have made the freshly cut apples at 0 h and after 1 h consistent in their surface states in order to have achieved browning inhibition.

A total of 122 statistically significant DAMs were identified among CK_0h vs. T_0h, of which, 52 were upregulated and 70 were downregulated (**Figure 7C**; **Supplementary Table 9**). For CK_1h vs. T_1h, 111 metabolites were significantly identified, with 46 significantly upregulated and 65 downregulated (**Figure 7D**; **Supplementary Table 10**). In addition, there were 64 common differentially abundant metabolites in the two groups, 58 unique differentially abundant metabolites in CK_0h vs. T_0h, and 47 that were unique in the CK_1h vs. T_1h (**Figure 7B**). Among the DAMs detected in the CK_0h vs. T_0h and CK_1h vs. T_1h samples, the most enriched KEGG terms were starch and sucrose metabolism, phenylpropanoid biosynthesis, glycerophospholipid metabolism, flavonoid biosynthesis, and alanine, aspartate, and glutamate metabolism (**Figures 7E, F**). Among them, the results for the phenylpropanoid biosynthesis, flavonoid biosynthesis, and alanine, aspartate, and glutamate metabolism pathways were consistent with the transcriptional results. Both phenylpropanoids and flavonoids are phenolic compounds, which means that the most differentially abundant metabolites in the browning process were phenolic compounds. In view of the role of phenolic compounds in improving plant antioxidant capacity, we inferred that the low degree of browning observed in the freshly cut apples in group T may be related to the high enrichment of these phenolic substances. Because the Maillard reaction is a natural reaction involving amino compounds (amines, amino acids, peptides, and proteins) and carbonyl compounds (reducing sugars) in food during its processing and storage, it is speculated that alanine, aspartate, and glutamate metabolism is related to non-enzymatic browning.

### 3.6 Analysis of differentially abundant metabolites during the browning of “Fuji” apples

**Figure 8:**
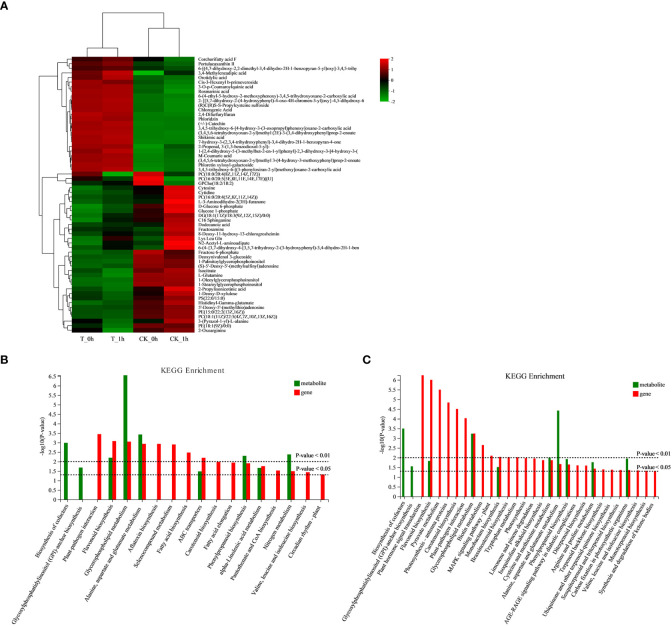
**(A)** Cluster heatmap of differential abundant metabolites in fresh-cut apple fruits. **(B)** Histograms of joint KEGG enrichment *p*-values in CK_0h vs. T_0h. **(C)** Histograms of joint KEGG enrichment *p*-values in CK_1h vs. T_1h.

We conducted a cluster analysis of the DAMs and selected the first 60 metabolites to construct a cluster heatmap, which could be divided into four categories according to the changing trend of the differential metabolites. In type I, the content of phenolic substances in the control and Se treatment groups changed significantly (**Figure 8A**; **Supplementary Table 11**). Among them, the expression of several phenolic substances, such as rosmarinic acid, chlorogenic acid, phloridzin, catechin, shikimic acid, M-coumaric acid, and phloretin xylosyl-galactoside, was significantly higher in T_0h and T_1h compared to CK_0h and CK_1h, while during the browning period between 0 h to 1 h after cutting, the phenolic contents showed no significant difference. The other three types of DAMs were found to be mainly involved in glycerophospholipid and fatty acid metabolism, among which, three metabolites were significantly upregulated in CK_0h [PC (18:0/20:4 (8Z,11Z,14Z,17Z)), PC (16:0/20:5 (5E,8E,11E,14E,17E)) [U], and GPCho (18:2/18:2)]. PC (16:0/20:4 (5Z,8Z,11Z,14Z)), glucose 1-phosphate, DG (18:1 (11Z)/18:3 (9Z,12Z,15Z)/0:0), dodecanoic acid, and 8-deoxy-11-hydroxy-13-chlorogrosheimin were significantly upregulated in CK_1h, while the rest were upregulated in the control and downregulated in the exogenous Se treatment samples.

The co-joint KEGG enrichment analysis revealed that there were seven co-mapped pathways in CK_0h vs. T_0h and eight in CK_1h vs. T_1h; of these, those that were enriched included flavonoid biosynthesis, phenylpropanoid biosynthesis, and glycerophospholipid metabolism (**Figures 8B, C**). The results of the joint analysis are consistent with the results produced by the above analyses of the DEGs and DAMs.

**Figure 9:**
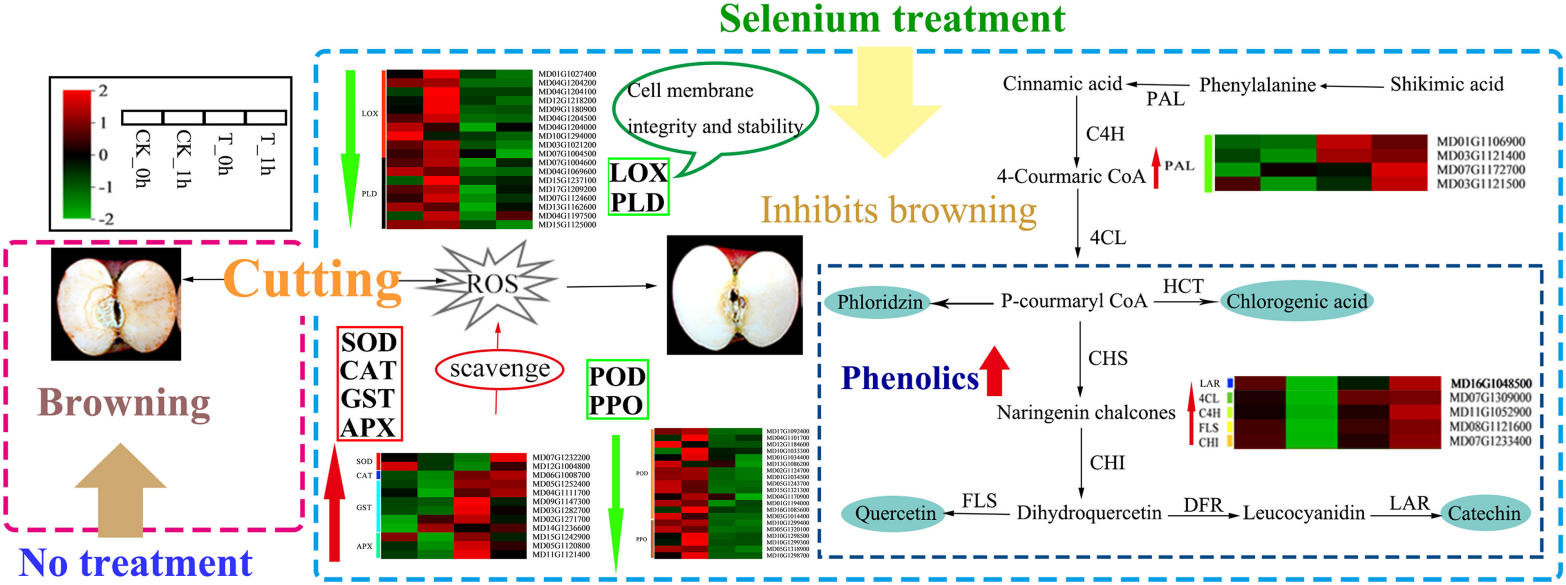
Proposed model for the browning inhibition of fresh-cut apples by exogenous Se. Red and green arrows indicate increased and decreased levels of genes and metabolites associated with control and exogenous Se treatment, respectively.

In summary, we have briefly described the potential mechanism by which exogenous Se inhibits fresh-cut apple browning (**Figure 9**). The present study found that exogenous Se treatment could reduce the activity of phenolase (PPO and POD activity) and inhibit the activities of LOX and PLD to help maintain the integrity and stability of the cell membrane. The transcriptomic results also showed that the expression levels of browning-related genes (such as *PPO*, *POD*, *LOX*, and *PLD*) were inhibited, indicating that exogenous Se inhibited enzymatic browning. Furthermore, exogenous Se enhanced several antioxidant enzymes (*SOD*, *CAT*, *APX*, and *GST*) and non-enzymatic (phenolic) ROS scavenging systems. The increases in the activities and gene expression levels of *SOD*, *CAT*, *APX*, and *GST* contribute to the removal of ROS accumulation in plants, thereby helping to maintain the dynamic balance of intracellular ROS. The increase in the phenolic contents of the fruit is inferred to have played a positive role in scavenging excessive free radicals. These results provide us with a new understanding of how exogenous Se inhibits the browning of freshly cut apple fruits.

## 4 Discussion

The browning of freshly cut apples has long been a major negative influencing factor on their flavor, nutrition, shelf life, and marketability. Surface browning occurs due to PPO catalyzing the oxidation of polyphenols to form quinones, which are then condensed into dark brown substances (Degl'Innocenti et al., 2005). POD is also a phenol oxidase, which can combine with H_2_O_2_ and catalyze the oxidation of phenols to free radicals. The inhibition of *PPO* gene expression is reported to be an effective method to reduce browning in potato tubers and *Agaricus bisporus* (Chi et al., 2014; Lei et al., 2018). Similarly, He et al. (2020) showed that synephrine hydrochloride (Syn-HCl) inhibited the browning of litchi pericarp by modulating POD and PPO activities. Liu et al. (2021) carried out a transcriptional analysis on freshly cut eggplant fruit, including cultivars that were browning-sensitive and browning-resistant. The results showed that the expression levels of *PPO* and *POD* genes were significantly upregulated in the browning-sensitive cultivar when compared to the browning-resistant cultivar at 15 min after cutting. In the present study, the gene expression levels of *POD* and *PPO* were more significantly upregulated in the control compared to the exogenous Se treatment group (**Figure 4A**; **Supplementary Table 7**); the *POD* genes were more highly expressed at either 0 h or 1 h compared to the exogenous Se treatment group, whereas the *PPO* genes were upregulated in the control at 1 h relative to 0 h, but they did not exhibit differential expression in the samples subjected to exogenous Se treatment. **Figures 5A, B** also show that the activities of PPO and POD were higher in the control compared to the exogenous Se treatment group. Therefore, PPO and POD may play a synergistic role during the browning process of freshly cut apples.

Phenolic substances act as substrates during their involvement in enzymatic browning. The antioxidant activities of polyphenols, on the other hand, function to eliminate excessive oxygen free radicals in cells and maintain the cell redox balance (Robards et al., 1999; Venturi et al., 2019). The metabolome results of the present study showed that exogenous Se treatment induced the synthesis and accumulation of several common phenolics, such as rosmarinic acid, chlorogenic acid, phloridzin, catechin, shikimic acid, and M-coumaric acid, all of which were significantly higher in the exogenous Se treatment samples compared to the control samples (**Figure 8A**). Xu et al. (2021) reported that the expression levels of polyphenols, such as catechin, quercetin, and phloretin, decreased in the browned tissues of apples, contributing to a decrease in intracellular antioxidant substances involved in scavenging ROS. This finding was further supported by transcriptional analysis (**Figure 4A**), which demonstrated that the expression levels of the *PAL*, *4CL*, *C4H*, *FLS*, and *CHI* genes involved in the phenylpropanoid and flavonoid biosynthetic pathways were upregulated. A study on freshly cut sweet potatoes treated with ultrasound found that the induction of PAL was positively correlated with higher total phenolic content, thereby enhancing the antioxidant capacity of the freshly cut sweet potatoes to protect them against browning (Pan et al., 2020). It has also been reported that 80% oxygen treatment could increase the total phenol content of freshly cut potatoes, effectively enhancing their antioxidant capacity (Liu et al., 2019).

The browning of freshly cut apples is caused by the destruction of the cellular regional spatial structure of phenolic substances and enzymes during the cutting process. Li et al. (2017) found that cell membrane stability played an important role in inhibiting the browning of freshly cut pears. Lipids are important components of cell membranes; the reduction of unsaturated fatty acids or the degradation of membrane phospholipids can alter cell membrane permeability and impair membrane integrity (Saquet et al., 2003; Zhang et al., 2018). LOX and PLD are two important lipid-degrading enzymes; the former induces the oxidation of unsaturated fatty acids to produce peroxidation products, while the latter induced the degradation of phospholipids in lipase to free fatty acids (Yi et al., 2008; Wang et al., 2013; Lin et al., 2016). These peroxidation products subsequently damage the cell membrane and trigger the onset of the enzymatic browning reaction (Zhang et al., 2010; Cai et al., 2015). From this study, it is clear that exogenous Se treatment may reduce the activity of membrane lipid-degrading enzymes by inhibiting the expression levels of the *LOX* and *PLD* genes (**Figure 4A**), thereby delaying the degradation of cell membrane lipids and maintaining the integrity of the cell membrane structure. By measuring physiological indexes, it was found that the LOX and PLD activities of freshly cut apples after exogenous Se treatment were significantly lower than those of the control (**Figures 5C, D**). The metabolic analysis also showed that DAMs involved in glycerophospholipid and fatty acid metabolism were upregulated in the control, while exogenous Se treatment inhibited the expression of these metabolites (**Figure 8A**). A previous study found that elevated CO_2_ treatment delayed membrane lipid peroxidation and maintained membrane integrity by inhibiting the activities of LOX and PLD, thereby inhibiting the occurrence of browning (Wang et al., 2021). Song et al. (2022) also reported that hot air treatment inhibited the browning of pineapple fruit during storage by inhibiting the expression levels of the *PLD* and *LOX* genes. Similar reports have been given for apples (Chen C. et al., 2021), cherries (Rabiei et al., 2019), and lotus seeds (Luo et al., 2020).

Mechanical damage caused by fruit cutting increases ROS production, which accelerates membrane lipid peroxidation and promotes browning. The enzymatic antioxidant system plays an important role in controlling ROS production and maintaining cellular redox homeostasis (Li et al., 2022). Antioxidant enzymes include SOD, CAT, APX, and GST (Wu et al., 2012). In the present study, genes encoding antioxidant enzyme activities, such as SOD, CAT, APX, and GST, were found to have been upregulated in the T group when compared to the control. Another study found that the expression levels of 18 GST-coding genes and 10 APX-coding genes were significantly higher in a freshly cut eggplant browning-resistant cultivar when compared with a browning-sensitive cultivar (Liu et al., 2021). Similarly, browning-resistant bananas treated with glycine betaine (GB) exhibited relatively higher expression levels of *MaAPX*, *MaCAT*, and *MaSOD* when compared to browning-prone control fruit, while GB treatment could significantly enhance their total antioxidant capacity (Chen LL. et al., 2021). The expression of GST increases, resulting in an increase in the reducing agent glutathione in cells; in turn, this leads to an increase in the production of antioxidants in cells, which ultimately remove ROS in cells to reduce browning (Liu et al., 2022). Se is involved in the formation of glutathione peroxidase (GSH-Px) activity centers, which protect the cell membrane by participating in redox homeostasis within cells (Kieliszek and Błażejak, 2013; Kieliszek and Błażejak, 2016). Li et al. (2020) reported that core-browning in late-harvested “Yali” pear fruit was more likely to occur than that in mid-season-harvested “Yali” fruit because the late-harvested fruit contained lower SOD and CAT activities. Hou et al. (2022) found that the higher browning resistance that is characteristic of the “Shannongsu” pear may be related to its higher CAT activity. The increases in SOD and CAT activities facilitate the removal of ROS and reduce oxidative damage in cells, converting H_2_O_2_ into water in the process (Abdelhai et al., 2019; Li et al., 2019). Studies have shown that freshly cut apples with higher antioxidant capacity exhibit a lesser degree of browning (Aguayo et al., 2010; Saba and Sogvar, 2016; Putnik et al., 2017), and our results support this. Therefore, we believe that the anti-browning effect of exogenous Se treatment in freshly cut apples may be related to its ability to enhance the antioxidant capacity of the fruits and hence reduce the oxidative damage caused by ROS to cell membranes.

Several transcription factors, such as MYB, WRKY, and ERF, are associated with oxidative stress responses, including those caused by physical trauma. Previous studies have shown that the WRKY and MYB transcription factors play comparable roles to PPO in the regulation of apple browning (Zuo et al., 2021). The higher ERF transcription factor expression pattern observed in the control in the current study was consistent with a previous study on browning in freshly cut lotus root (Qiao et al., 2022). In addition, other families of transcription factors, such as HB-other, NAC, and BHLH, also showed different levels of transcription. Changes in these transcription factors may also be related to the browning process of freshly cut apples (**Figure 4B**). However, their functional properties will need to be further explored to clarify their potential impact on the browning of freshly cut apples.

## 5 Conclusion

The results herein show that the browning of freshly cut apples treated with exogenous Se was significantly lower than that of the control apples. This indicates that the use of exogenous Se represents a novel and effective method to inhibit the browning of postharvest apples during apple cultivation (**Figure 9**). The expression levels of the *PPO* and *POD* genes in the control sample were higher than those in the Se treatment group, while the phenolic contents showed the opposite results, indicating that exogenous Se inhibited apple browning by modulating phenolase activity. In addition, the differential gene expression of *LOX* and *PLD* involved in fatty acid and lipid biosynthesis pathways in the exogenously Se-treated samples was significantly higher compared to controls, which provides more evidence that membrane stability is a potential factor that affects the degree of fruit browning. Furthermore, the expression levels of genes related to antioxidant enzymes (SOD, CAT, GST, and APX) were upregulated in the treatment group, indicating that the browning-inhibition mechanism by which exogenous Se acts on freshly cut apples may be the result of the joint action of multiple metabolic pathways.

## Data availability statement

The data presented in the study are deposited in the National Library of Medicine, National Center for Biotechnology Information repository, accession number PRJNA953812.

## Author contributions

XW and XZ performed writing—original draft. PJ performed investigation. HAL, GQ, and XZ performed data curation. HL, SG, and XZ contributed to resources. XW and XZ contributed to methodology. XZ performed funding acquisition and writing—review, and editing. All authors contributed to the article and approved the submitted version.

## References

[B1] Abdelhai M. H. Zhang Q. Zhao L. Mahunu G. K. Musa A. Yang Q.. (2019). Effects of baobab *(Adansonia digitata* l.) in combination with *Sporidiobolus pararoseus* Y16 on the activities of the defense-related enzymes and the expression levels of defense-related genes of apples. Biol. Control 139, 104094. doi: 10.1016/j.biocontrol.2019.104094

[B2] Aguayo E. Requejo-Jackman C. Stanley R. Woolf A. (2010). Effects of calcium ascorbate treatments and storage atmosphere on antioxidant activity and quality of fresh-cut apple slices. Postharvest Biol. Technol. 57, 52–60. doi: 10.1016/j.postharvbio.2010.03.001

[B3] Bondonno N. P. Bondonno C. P. Ward N. C. Hodgson J. M. Croft K. D. (2017). The cardiovascular health benefits of apples: whole fruit vs. isolated compounds. Trends Food Sci. Technol. 69, 243–256. doi: 10.1016/j.tifs.2017.04.012

[B4] Boyer J. Liu R. H. (2004). Apple phytochemicals and their health benefits. Nutr. J. 3, 1–15. doi: 10.1186/1475-2891-3-5 15140261 PMC442131

[B5] Cai J. H. Chen J. Lu G. B. Zhao Y. M. Tian S. P. Qin G. Z. (2015). Control of brown rot on jujube and peach fruits by trisodium phosphate. Postharvest Biol. Technol. 99, 93–98. doi: 10.1016/j.postharvbio.2014.08.003

[B6] Chen C. Jiang A. L. Liu C. H. Wagstaff C. Zhao Q. Q. Zhang Y. H.. (2021). Hydrogen sulfide inhibits the browning of fresh-cut apple by regulating the antioxidant, energy and lipid metabolism. Postharvest Biol. Technol. 175, 111487. doi: 10.1016/j.postharvbio.2021.111487

[B7] Chen L. L. Shan W. Cai D.l. Chen J. Y. Lu W. J. Su X. G.. (2021). Postharvest application of glycine betaine ameliorates chilling injury in cold-stored banana fruit by enhancing antioxidant system. Sci. Hortic. 287, 110264. doi: 10.1016/j.scienta.2021.110264

[B8] Chi M. Bhagwat B. Lane W. D. Tang G. L. Shu Y. Q. Sun R. C.. (2014). Reduced polyphenol oxidase gene expression and enzymatic browning in potato *(Solanum tuberosum* l.) with artificial microRNAs. BMC Plant Biol. 14, 62. doi: 10.1186/1471-2229-14-62 24618103 PMC4007649

[B9] Degl'Innocenti E. Guidi L. Pardossi A. TognoniD F. (2005). Biochemical study of leaf browning in minimally processed leaves of lettuce *(Lactuca sativa* l. var. *acephala*). J. Agric. Food Chem. 53, 9980–9984. doi: 10.1021/jf050927o 16366683

[B10] Fan X. T. (2022). Chemical inhibition of polyphenol oxidase and cut surface browning of fresh-cut apples. Crit. Rev. Food Sci. Nutr. 13, 1–15. doi: 10.1080/10408398.2022.2061413 35416745

[B11] Hamdan N. Lee C. H. Wong S. L. Fauzi C. Zamri N. M. A. Lee T. H. (2022). Prevention of enzymatic browning by natural extracts and genome-editing: a review on recent progress. Molecules 27, 1101. doi: 10.3390/molecules27031101 35164369 PMC8839884

[B12] Hao P. P. Wang G. M. Cheng H. Y. Ke Y. Q. Qi K. J. Gu C.. (2018). Transcriptome analysis unravels an ethylene response factor involved in regulating fruit ripening in pear. Physiol. Plant 163, 124–135. doi: 10.1111/ppl.12671 29148054

[B13] He M. Y. Ge Z. X. Hong M. Qu H. X. Duan X. W. Yun Z.. (2020). Alleviation of pericarp browning in harvested litchi fruit by synephrine hydrochloride in relation to membrane lipids metabolism. Postharvest Biol. Technol. 166, 111223. doi: 10.1016/j.postharvbio.2020.111223

[B14] Hou X. K. Liu W. J. Zuo W. F. Zhang R. Zou Q. Zhang S. S.. (2022). Analysis of enzymes and phenolic metabolites which affecting the anti-browning property of ‘Shannongsu’pear. LWT 168, 113919. doi: 10.1016/j.lwt.2022.113919

[B15] Hu W. Z. Guan Y. G. Ji Y. R. Yang X. Z. (2021). Effect of cutting styles on quality, antioxidant activity, membrane lipid peroxidation, and browning in fresh-cut potatoes. Food Biosci. 44, 101435. doi: 10.1016/j.fbio.2021.101435

[B16] Imahori Y. (2014). “"Role of ascorbate peroxidase in postharvest treatments of horticultural crops",” in Oxidative damage to plants. Eds. Ahmad P. Prasad M. N. V. (San Diego, CA, USA: Academic Press), 425–451.

[B17] Kähkönen M. P. Hopia A. I. Vuorela H. J. Rauha J.-P. Pihlaja K. Kujala T. S.. (1999). Antioxidant activity of plant extracts containing phenolic compounds. J. Agric. Food Chem. 47, 3954–3962. doi: 10.1021/jf990146l 10552749

[B18] Kamran M. Parveen A. Ahmar S. Malik Z. Hussain S. Chattha M. S.. (2019). An overview of hazardous impacts of soil salinity in crops, tolerance mechanisms, and amelioration through selenium supplementation. Int. J. Mol. Sci. 21, 148. doi: 10.3390/ijms21010148 31878296 PMC6981449

[B19] Kieliszek M. Błażejak S. (2013). Selenium: significance, and outlook for supplementation. Nutrition 29, 713–718. doi: 10.1016/j.nut.2012.11.012 23422539

[B20] Kieliszek M. Błażejak S. (2016). Current knowledge on the importance of selenium in food for living organisms: a review. Molecules 21, 609. doi: 10.3390/molecules21050609 27171069 PMC6274134

[B21] Kong X. M. Ge W. Y. Wei B. D. Zhou Q. Zhou X. Zhao Y. B.. (2020). Melatonin ameliorates chilling injury in green bell peppers during storage by regulating membrane lipid metabolism and antioxidant capacity. Postharvest Biol. Technol. 170, 111315. doi: 10.1016/j.postharvbio.2020.111315

[B22] Korkman J. (1980). The effect of selenium fertilizers on the selenium content of barley spring wheat and potatoes. Agr. Food Sci. 52, 495–504. doi: 10.23986/AFSCI.72049

[B23] Lei J. Li B. J. Zhang N. Yan R. X. Guan W. Q. Brennan C. S.. (2018). Effects of UV-c treatment on browning and the expression of polyphenol oxidase (PPO) genes in different tissues of *Agaricus bisporus* during cold storage. Postharvest Biol. Technol. 139, 99–105. doi: 10.1016/j.postharvbio.2017.11.022

[B24] Li H. Fan Y. W. Zhi H. Zhu Y. Y. Liu Y. G. Wang Y. S. (2019). Influence of fruit stalk on reactive oxygen species metabolism and quality maintenance of peach fruit under chilling injury condition. Postharvest Biol. Technol. 148, 141–150. doi: 10.1016/j.postharvbio.2018.10.018

[B25] Li L. Zhang Y. Y. Fan X. L. Wang J. D. Liang L. Y. Yan S. J.. (2020). Relationship between activated oxygen metabolism and browning of “Yali” pears during storage. J. Food Process. Preserv. 44, e14392. doi: 10.1111/jfpp.14392

[B26] Li Z. H. Zhang Y. X. Ge H. B. (2017). The membrane may be an important factor in browning of fresh-cut pear. Food Chem. 230, 265–270. doi: 10.1016/j.foodchem.2017.03.044 28407910

[B27] Li Y. X. Zhang L. Zhang L. Nawaz G. Zhao C. X. Zhang J.. (2022). Exogenous melatonin alleviates browning of fresh-cut sweetpotato by enhancing anti-oxidative process. Sci. Hortic. 297, 110937. doi: 10.1016/j.scienta.2022.110937

[B28] Liao X. L. Rao S. Yu T. Zhu Z. Z. Yang X. Y. Xue H.. (2021). Selenium yeast promoted the Se accumulation, nutrient quality and antioxidant system of cabbage *(Brassica oleracea* var. *capitata* l.). Plant Signal. Behav. 16, 1907042. doi: 10.1080/15592324.2021.1907042 33818289 PMC8143226

[B29] Lin Y. F. Lin H. T. Lin Y. X. Zhang S. Chen Y. H. Jiang X. ,. J. (2016). The roles of metabolism of membrane lipids and phenolics in hydrogen peroxide-induced pericarp browning of harvested longan fruit. Postharvest Biol. Technol. 111, 53–61. doi: 10.1016/j.postharvbio.2015.07.030

[B30] Liu X. Wang T. Lu Y. Z. Yang Q. Li Y. Deng X. D.. (2019). Effect of high oxygen pretreatment of whole tuber on anti-browning of fresh-cut potato slices during storage. Food Chem. 301, 125287. doi: 10.1016/j.foodchem.2019.125287 31387048

[B31] Liu L. Wang Z. M. Niu C. Q. Yuan L. X. Liu J. Liu Z. K.. (2020). Effects of soil applied selenium on selenium content and quality of ‘Gala’ apple fruits. China Fruits 201, 27–30+35. doi: 10.16626/j.cnki.issn1000-8047.2020.01.006

[B32] Liu X. H. Xiao K. Zhang A. D. Zhu W. M. Zhang H. Tan F.. (2022). Metabolomic analysis, combined with enzymatic and transcriptome assays, to reveal the browning resistance mechanism of fresh-cut eggplant. Foods 11, 1174. doi: 10.3390/foods11081174 35454761 PMC9031582

[B33] Liu X. H. Zhang A. D. Zhao J. Shang J. Zhu Z. W. Wu X. X.. (2021). Transcriptome profiling reveals potential genes involved in browning of fresh-cut eggplant *(Solanum melongena* l.). Sci. Rep. 11, 16081. doi: 10.1038/S41598-021-94831-Z 34373468 PMC8352891

[B34] Livak K. J. Schmittgen T. D. (2001). Analysis of relative gene expression data using real-time quantitative PCR and the 2^–ΔΔCT^ method. Methods 25, 402–408. doi: 10.1006/meth.2001.1262 11846609

[B35] Luo S. F. Hu H. L. Wang Y. Zhou H. S. Zhang Y. T. Zhang L. G.. (2020). The role of melatonin in alleviating the postharvest browning of lotus seeds through energy metabolism and membrane lipid metabolism. Postharvest Biol. Technol. 167, 111243. doi: 10.1016/j.postharvbio.2020.111243

[B36] Min T. Bao Y. Q. Zhou B. X. Yi Y. Wang L. M. Hou W. F.. (2019). Transcription profiles reveal the regulatory synthesis of phenols during the development of lotus rhizome *(Nelumbo nucifera* gaertn). Int. J. Mol. Sci. 20, 2735. doi: 10.3390/ijms20112735 31167353 PMC6600570

[B37] Mishra B. B. Gautam S. Sharma A. (2012). Browning of fresh-cut eggplant: impact of cutting and storage. Postharvest Biol. Technol. 67, 44–51. doi: 10.1016/j.postharvbio.2011.12.009

[B38] Moon K. M. Kwon E. B. Lee B. Kim C. Y. (2020). Recent trends in controlling the enzymatic browning of fruit and vegetable products. Molecules 25, 2754. doi: 10.3390/molecules25122754 32549214 PMC7355983

[B39] Nielsen F. H. (1990). New essential trace elements for the life sciences. Biol. Trace Elem. Res. 26, 599–611. doi: 10.1007/BF02992716 1704767

[B40] Pan Y. F. Chen L. Pang L. L. Chen X. T. Jia X. T. Li X. H. (2020). Ultrasound treatment inhibits browning and improves antioxidant capacity of fresh-cut sweet potato during cold storage. RSC Adv. 10, 9193–9202. doi: 10.1039/C9RA06418D 35497218 PMC9050142

[B41] Putnik P. Bursać Kovačević D. Herceg K. Pavkov I. Zorić Z. Levaj B. (2017). Effects of modified atmosphere, anti-browning treatments and ultrasound on the polyphenolic stability, antioxidant capacity and microbial growth in fresh-cut apples. J. Food Process Eng. 40, e12539. doi: 10.1111/jfpe.12539

[B42] Qi X. X. (2017). Research advance in enzymatic browning and inhibition method of postharvest of fruits and vegetable. North. Hortic. 386, 190–194. doi: 10.11937/bfyy.201711040

[B43] Qi X. C. Jian Z. H. Zhang Q. Song S. W. (2019). Effects of foliar application of selenium on selenium and heavy metal contents and fruit quality in sweet cherry. J. Fruit Sci. 36, 748–754. doi: 10.13925/j.cnki.gsxb.20180505

[B44] Qiao L. P. Gao M. Wang Y. S. Tian X. ,. J. Lu L. F. Liu X. (2022). Integrated transcriptomic and metabolomic analysis of cultivar differences provides insights into the browning mechanism of fresh-cut potato tubers. Postharvest Biol. Technol. 188, 111905. doi: 10.1016/j.postharvbio.2022.111905

[B45] Rabiei V. Kakavand F. Zaare-Nahandi F. Razavi F. Aghdam M. S. (2019). Nitric oxide and γ-aminobutyric acid treatments delay senescence of cornelian cherry fruits during postharvest cold storage by enhancing antioxidant system activity. Sci. Hortic. 243, 268–273. doi: 10.1016/j.scienta.2018.08.034

[B46] Robards K. Prenzler P. D. Tucker G. Swatsitang P. Glover W. (1999). Phenolic compounds and their role in oxidative processes in fruits. Food Chem. 66, 401–436. doi: 10.1016/S0308-8146(99)00093-X

[B47] Saba M. K. Sogvar O. B. (2016). Combination of carboxymethyl cellulose-based coatings with calcium and ascorbic acid impacts in browning and quality of fresh-cut apples. LWT-Food Sci. Technol. 66, 165–171. doi: 10.1016/j.lwt.2015.10.022

[B48] Saquet A. A. Streif J. Bangerth F. (2003). Energy metabolism and membrane lipid alterations in relation to brown heart development in ‘Conference’ pears during delayed controlled atmosphere storage. Postharvest Biol. Technol. 30, 123–132. doi: 10.1016/s0925-5214(03)00099-1

[B49] Singh B. Suri K. Shevkani K. Kaur A. Kaur A. Singh N. (2018). “"Enzymatic browning of fruit and vegetables: a review",” in Enzymes in food technology, vol. . p . Ed. Kuddus M. (Singapore: Springer), 63–78. doi: 10.1007/978-981-13-1933-4_4

[B50] Song K. Gu H. Golding J. B. Pristijono P. Hou X. W. Zhang L. B.. (2022). Insight into the physiological and molecular mechanisms of hot air treatment which reduce internal browning in winter-harvested pineapples. Postharvest Biol. Technol. 194, 112066. doi: 10.1016/j.postharvbio.2022.112066

[B51] Sumonsiri N. (2017). Effect of ascorbicacid and nisin on fresh-cut apples. Carpathian J. Food Sci. Technol. 9, 71–85. https://www.researchgate.net/publication/322736781

[B52] Tang T. T. Xie X. F. Ren X. Wang W. J. Tang X. M. Zhang J.. (2020). A difference of enzymatic browning unrelated to PPO from physiology, targeted metabolomics and gene expression analysis in Fuji apples. Postharvest Biol. Technol. 170, 111323. doi: 10.1016/j.postharvbio.2020.111323

[B53] Tang C. H. Zhao Q. Y. Zhang K. Li S. Qin Y. C. Zhang J. M. (2019). Promoting the development of nutritionally-guided agriculture in research and development of selenium-enriched agri-products in China. Scientia Agric. Sin. 52, 3122–3133. doi: 10.3864/j.issn.0578-1752.2019.18.005

[B54] Ullah H. Liu G. Yousaf B. Ali M. U. Irshad S. Abbas Q.. (2019). A comprehensive review on environmental transformation of selenium: recent advances and research perspectives. Environ. Geochem. Health 41, 1003–1035. doi: 10.1007/s10653-018-0195-8 30267320

[B55] Venturi F. Bartolini S. Sanmartin C. Orlando M. Taglieri I. Macaluso M.. (2019). Potato peels as a source of novel green extracts suitable as antioxidant additives for fresh-cut fruits. Appl. Sci. 9, 2431. doi: 10.3390/app9122431

[B56] Wang J. W. Jiang Y. A. Li G. D. Lv M. Zhou X. Zhou Q.. (2018). Effect of low temperature storage on energy and lipid metabolisms accompanying peel browning of ‘Nanguo’pears during shelf life. Postharvest Biol. Technol. 139, 75–81. doi: 10.1016/j.postharvbio.2018.01.020

[B57] Wang D. Li D. Xu Y. Q. Li L. Belwal T. Zhang X. C.. (2021). Elevated CO_2_ alleviates browning development by modulating metabolisms of membrane lipids, proline, and GABA in fresh-cut Asian pear fruit. Sci. Hortic. 281, 109932. doi: 10.1016/j.scienta.2021.109932

[B58] Wang X. Y. Li H. R. Yang L. S. Kong C. Wang J. Li Y. C. (2020). Selenium nutritional status of rural residents and its correlation with dietary intake patterns in a typical low-selenium area in China. Nutrients 12, 3816. doi: 10.3390/nu12123816 33322199 PMC7764644

[B59] Wang H. Qian Z. J. Ma S. M. Zhou Y. C. Patrick J. W. Duan X. W.. (2013). Energy status of ripening and postharvest senescent fruit of litchi *(Litchi chinensis* sonn.). BMC Plant Biol. 13, 1–16. doi: 10.1186/1471-2229-13-55 23547657 PMC3636124

[B60] Wen B. Li D. Tang D. Huang Z. Kedbanglai P. Ge Z. B.. (2020). Effects of simultaneous ultrasonic and cysteine treatment on antibrowning and physicochemical quality of fresh-cut lotus roots during cold storage. Postharvest Biol. Technol. 168, 111294. doi: 10.1016/j.postharvbio.2020.111294

[B61] Wu F. H. Yang H. Q. Chang Y. Z. Cheng J. Y. Bai S. F. X. Yin J. Y. (2012). Effects of nitric oxide on reactive oxygen species and antioxidant capacity in Chinese bayberry during storage. Sci. Hortic. 135, 106–111. doi: 10.1016/j.scienta.2011.12.011

[B62] Xiao Y. H. Xie J. Wu C. S. He J. M. Wang B. (2021). Effects of melatonin treatment on browning alleviation of fresh-cut foods. J. Food Biochem. 45, e13798. doi: 10.1111/jfbc.13798 34037249

[B63] Xu X. Y. Liu G. S. Li H. L. Tian H. Q. Fu D. Q. (2021). Analysis of apple postharment damage under high CO_2_ concentration by transcriptome combined with metabolome. Chin. J. Biotechnol. 37, 2856–2869. doi: 10.13345/j.cjb.200547 34472303

[B64] Xu Y. J. Wang D. Zhao W. T. Zheng Y. Y. Wang Y. B. Wang P.. (2022). Low frequency ultrasound treatment enhances antibrowning effect of ascorbic acid in fresh-cut potato slices. Food Chem. 380, 132190. doi: 10.1016/j.foodchem.2022.132190 35086012

[B65] Yang Y. J. Liu X. H. Ning C. J. Ji A. Q. Ding N. Xu L. J.. (2013). Effects of foliar feeding of selenium on fruit quality and accumulation of cadmium, lead and mercury in sweet persimmon. Acta Hortic. Sin. 40, 523–530. doi: 10.16420/j.issn.0513-353x.2013.03.016

[B66] Yi C. Qu H. X. Jiang Y. M. Shi J. Duan X. W. Joyce D. C.. (2008). ATP-induced changes in energy status and membrane integrity of harvested litchi fruit and its relation to pathogen resistance. J. Phytopathol. 156, 365–371. doi: 10.1111/j.1439-0434.2007.01371.x

[B67] Yousuf B. Deshi V. Ozturk B. Siddiqui M. W. (2020). “Fresh-cut fruits and vegetables: quality issues and safety concerns,” in Fresh-cut fruits and vegetables. Ed. Siddiqui M. W. (Amsterdam, The Netherlands: Elsevier), 1–15. doi: 10.1016/B978-0-12-816184-5.00001-X

[B68] Zha Z. P. Tang R. Wang C. Li Y. L. Liu S. Wang L.. (2022). Riboflavin inhibits browning of fresh-cut apples by repressing phenolic metabolism and enhancing antioxidant system. Postharvest Biol. Technol. 187, 111867. doi: 10.1016/j.postharvbio.2022.111867

[B69] Zhang J. J. (2006). The biological functions of selenium and research development of Se-enriched foodstuff. Stud. Trace Elem. Health 3, 58–60. https://kns.cnki.net/kcms/detail/detail.aspx?FileName=WYJK200603026&DbName=CJFQ2006

[B70] Zhang C. F. Ding Z. S. Xu X. B. Wang Q. Qin G. Z. Tian S. P. (2010). Crucial roles of membrane stability and its related proteins in the tolerance of peach fruit to chilling injury. Amino Acids 39, 181–194. doi: 10.1007/s00726-009-0397-6 20091071

[B71] Zhang S. Lin Y. Z. Lin H. T. Lin Y. X. Chen Y. H. Wang H.. (2018). *Lasiodiplodia theobromae* (Pat.) griff. & maubl.-induced disease development and pericarp browning of harvested longan fruit in association with membrane lipids metabolism. Food Chem. 244, 93–101. doi: 10.1016/j.foodchem.2017.10.020 29120810

[B72] Zhou H. S. Yin H. Chen J. Q. Liu X. Gao Y. B. Wu J. Y.. (2016). Gene-expression profile of developing pollen tube of *Pyrus bretschneideri* . Gene Expr. Patterns 20, 11–21. doi: 10.1016/j.gep.2015.10.004 26547040

[B73] Zhu L. Q. Wei Q. P. Xu X. F. Han Z. H. Wang X. W. Li T. Z. (2007). Selenium absorbtion, distribution and accumulation in grapevine. Acta Hortic. Sin. 34, 325–328. doi: 10.16420/j.issn.0513-353x.2007.02.012

[B74] Zuo W. F. Lu L. Su M. Y. Zhang J. Li Y. Y. Huang S. L.. (2021). Analysis of differentially expressed genes and differentially abundant metabolites associated with the browning of meihong red-fleshed apple fruit. Postharvest Bio. Technol. 174, 111437. doi: 10.1016/j.postharvbio.2020.111437

