# Supplementary material for: Transcriptomics and metabolomics provide insight into the anti-browning mechanism of selenium in freshly cut apples
Source: Front Plant Sci. 2023 May 8;14:1176936. doi: 10.3389/fpls.2023.1176936 (PMC10200898; doi:10.3389/fpls.2023.1176936)
Supplement: Supplementary file 1 [file DataSheet_1.zip › Supplementary_Material/Supplementary_Material.docx]

Supplementary Material

# Supplementary Tables

**Supplementary Table 1.** Comparison rate of sequencing results with apple genome.

**Supplementary Table 2.** Numbers of up- and down-regulated DEGs in CK_0h vs T_0h, CK_1h vs T_1h, CK_0h vs CK_1h, and T_0h vs T_1h.

**Supplementary Table 3.** The main GO functional categories in CK_0h and T_0h.

**Supplementary Table 4.**  The main GO functional categories in CK_1h and T_1h.

**Supplementary Table 5.** The KEGG enrichment pathway in CK_0h and T_0h.

**Supplementary Table 6.** The KEGG enrichment pathway in CK_1h and T_1h.

**Supplementary Table 7.** EDifferential expression of 59 browning-related genes under control and exogenous Se treatment.

**Supplementary Table 8.** Number of differentially expressed transcription factors.

**Supplementary Table 9.** DAMs in CK_0h vs T_0h.

**Supplementary Table 10.** DAMs in CK_1h vs T_1h.

**Supplementary Table 11.** The top 60 DAMs were selected for cluster analysis.

# Date sheet _1 and Date sheet _2: All raw data for the remaining figures.
